# Comprehensive mapping of the exterior architecture of the dromedary camel brain

**DOI:** 10.1038/s41598-024-53541-y

**Published:** 2024-02-05

**Authors:** Ahmad Al Aiyan, Rinsha Balan, Senit Gebreigziabiher, Simona Zerom, Yotam Mihreteab, Even Ghebrehiwot, Adnan AlDarwich, Arve Lee Willingham, Uday Kishore

**Affiliations:** Department of Veterinary Medicine, College of Agriculture and Veterinary Medicine, United Arab Emirates University, Al Ain, UAE

**Keywords:** Neuroscience, Zoology, Anatomy, Neurology

## Abstract

The morphological perspective of the camel brain remains largely unexplored. Therefore, studying the topography of the camel brain is of crucial importance. This study aimed to provide a detailed color-coded topographic representation of the camel brain's gross anatomy and nomenclature, showing its various gyri and sulci and their borders. We compared them to previously known information to develop a detailed description of camel brain exterior architecture. Our research identified distinctive gyri and sulci with discrete positions and surrounding structures, allowing us to define sulci boundaries and establish logical gyri nomenclature. This study uncovered previously overlooked gyri and sulci and improved descriptions of specific sulci. The ectomarginal sulcus, splenial sulcus, splenial gyrus, and ectogenual gyrus are a few examples. These findings highlight several unique anatomical features of the dromedary brain, which can guide future research. By providing a comprehensive examination of the distinctive exterior anatomical features of the camel brain, this study may serve as a point of convergence for all researchers, providing more accurate identification of the gyri and sulci.

## Introduction

Camels are valuable livestock species that live in semi-arid and arid regions and are known for their remarkable ability to survive thirst and hunger and thrive under the scorching sun due to their unique brain cooling mechanism, which protects the brain tissue and enables the animal to tolerate higher temperatures^1–3^. As a crucial component of the central nervous system, the brain plays a fundamental role in regulating various essential life functions^4^.

Despite the Dromedary camel species' distinctive ecological adaptations and socio-economic significance, our current understanding of its brain anatomy, particularly the external architecture, remains relatively limited. Particular interest lies in the layout and structure of the camel's cerebral cortex, characterized by a complex arrangement of folds known as gyri and grooves known as sulci. This intricate cortical landscape is crucial in various brain functions, including information processing, memory, consciousness, and motor function^5,6^.

Artiodactyls such as camels, cows, and giraffes are of particular interest in comparative neuroanatomy due to their brain size relative to non-human primates. When compared to primates and carnivores, the cerebral cortex of the artiodactyl exhibits even greater folding^7,8^.

Existing literature on the Dromedary camel's brain reveals only two studies examining the cortical gyri and sulci^9,10^. However, these studies presented partial accounts of the sulci and gyri of the camel brain. Moreover, the graphical representations offered in both studies are unclear, preventing a clear understanding of the camel brain’s exterior architecture. However, no substantial follow-up research has been conducted to explain these discrepancies or provide a clearer presentation of the gyri and sulci organization^9,10^.

Therefore, there is a clear and pressing need for a comprehensive, updated examination of the gyri and sulci of the Dromedary camel's brain to resolve these long-standing discrepancies and provide a deep understanding of its cortical architecture. This study will enhance our knowledge of camel neuroanatomy and contribute to our understanding of the functional implications of various cortical structures in ungulates and other mammalian groups, shedding light on their evolutionary adaptations.

The main goal of this study is to precisely construct a detailed and comprehensive map of the Dromedary camel's cerebrum, with a keen emphasis on understanding the complex organization of its gyri and sulci. Therefore, we aim to clarify the ambiguities from past studies and advance our understanding of this subject by providing precisely detailed, color-coded figures and delivering a high-resolution, accurate map of the Dromedary camel's cerebrum, which could act as a vital reference for subsequent neuroanatomical investigations. We aim to clarify the prior conflicting findings by presenting these figures from multiple perspectives, providing a novel and comprehensive understanding of the Dromedary camel's cerebral architecture. We attempt to carefully describe the gyri and sulci in terms of their precise locations, patterns, and dimensional characteristics. Additionally, we intend to conduct a comparative analysis of the cerebral architectures of other animal species. This will offer valuable insights into shared and neuroanatomical features and their evolutionary significance.

## Methods

In this study, we employed ethically approved and standardized methods to dissect 30 camel heads. We obtained 60 cerebral hemispheres from freshly slaughtered male dromedaries aged 2- 6 years, procured from the camel slaughterhouses of the Alain City Municipality, to analyze the exterior structure of the Dromedary brain. After removing the skin from the camels’ heads, we created a sizable opening in the skulls' dorsal wall, and the heads were subsequently immersed in containers filled with a 10% formaldehyde solution. The containers were then placed in a cold room at 5 °C for 1 month. Following this period, we carefully opened the skulls with surgical tools to expose the brains, which were then carefully extracted. The meninges covering the hemispheres' surface were removed, exposing the cerebral gyri and sulci, which we examined and photographed with a Sony a7R II camera. To examine the medial surface of the cerebral hemispheres, we employed a surgical blade to split the brain longitudinally along the longitudinal fissure. The captured cerebral gyri and sulci images were color-coded and labeled using Photoshop 2020 (version 21.1. 1). For all gyri and sulci identified in our study, we primarily adhered to the terminology set forth by the Nomina Anatomica Veterinaria^11^ guidelines and major related anatomy textbooks^12^. Additionally, we expanded our reference base with other camel publications^9,10,13^. To identify the boundaries of the gyri in the camel brain, we relied on the direction and extent of the presence of sulci and fissures, which allowed us to demarcate the boundaries between the gyrus, so the configuration and relationships of neighboring sulci provided additional context for boundary determination. Additionally, we used cerebral arterial branches to refine our identification of the cerebral sulci and gyri^3,14–16^.

## Results

**Figure 1:**
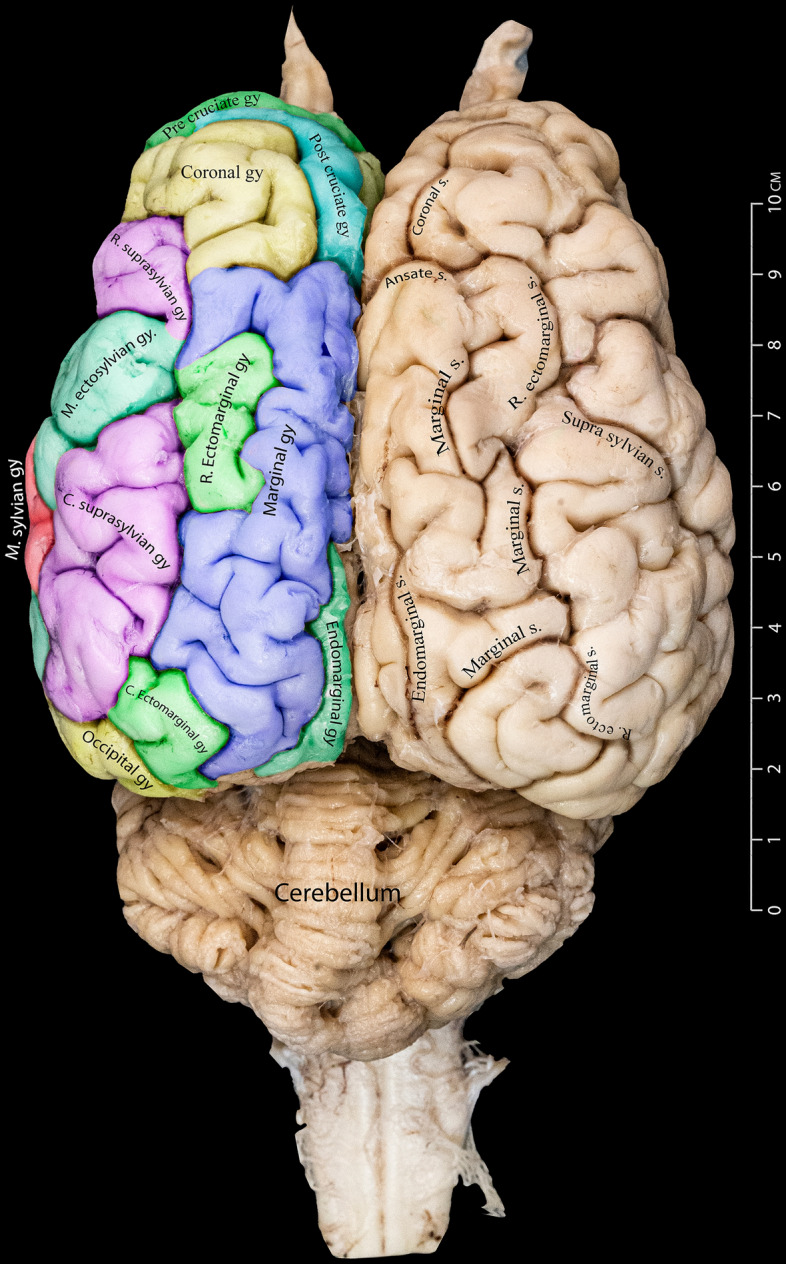
Dorsal view of the Dromedary camel brain. This figure provides a detailed representation of the gyri on the left cerebral hemisphere while highlighting the sulci on the right hemisphere.

This research aims to examine the external architecture of the dromedary camel brain, particularly its gross anatomy, from various perspectives, including dorsal, lateral, and medial views. To identify the precise locations, extensions, and dimensional characteristics of the sulci and gyri of the camel brain, we produced a color-coded camel brain showing the different gyri and sulci and their borders (Fig. 1).

### Sulci of the dromedary brain

The cortical surface of the camel’s brain hemisphere is complexly folded, and the main sulci exhibit a wide range of ramifications that may complicate their identification.

In our detailed examination of the cerebral hemispheres of the Dromedary camel, we observed a complex and distinct network of sulci. The intricate cerebral sulci that mark the brain's exterior and demarcate its gyri present a distinct pattern of cerebral folding.

### Sylvian fissure

**Figure 2:**
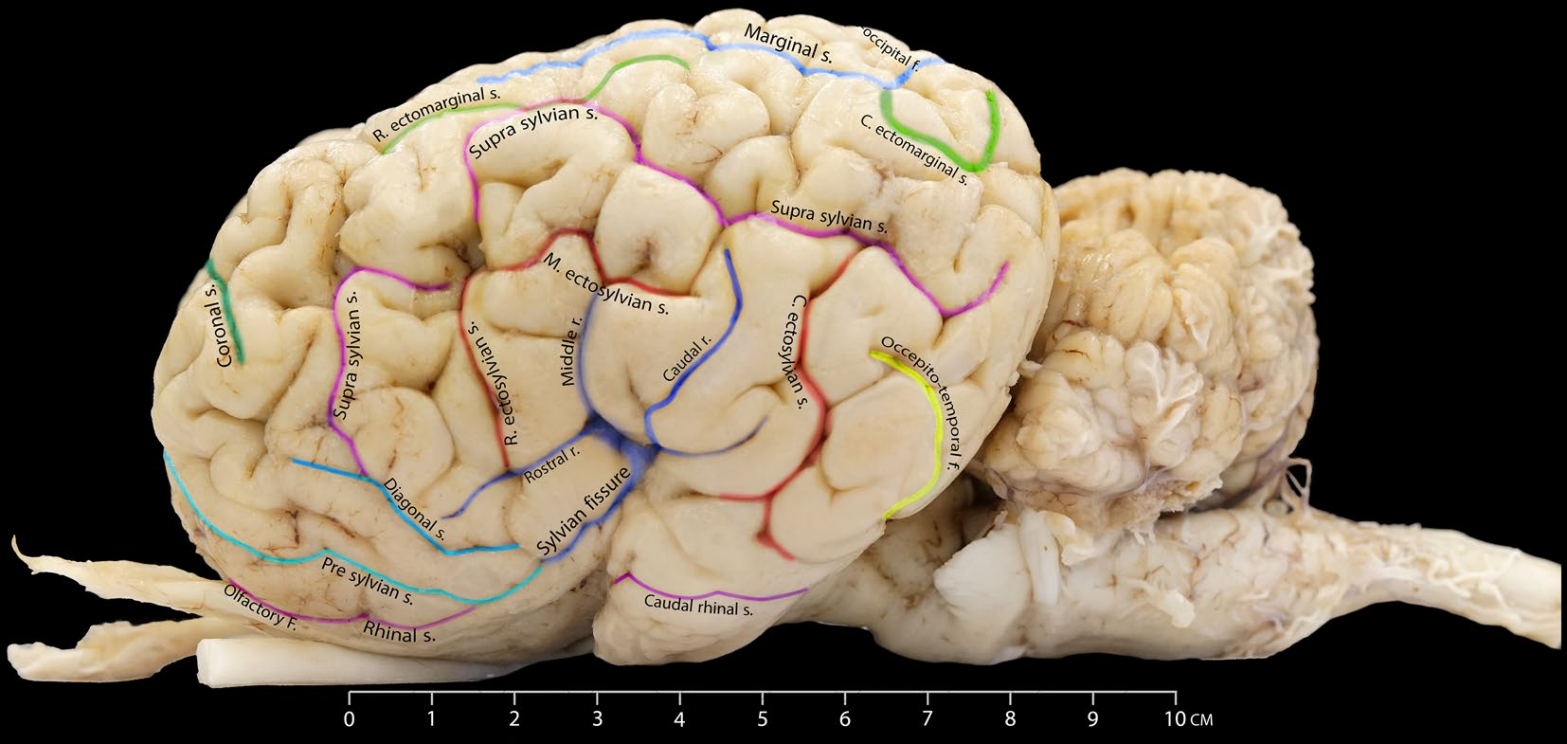
Detailed illustration of the various colored sulci on the lateral aspect of the dromedary camel's brain.

Our observations revealed that the sylvian fissure in the dromedary brain is the most prominent and deepest sulcus on the lateral surface of its hemisphere. It is short, almost vertical, arises ventrally near the piriform lobe, and runs dorsally, giving three main branches. The rostral limb extends rostrodorsally to the original sylvian fissure. The middle limb extends dorsally up to the level of the middle ectosylvian sulcus. Lastly, the caudal limb seems longer and runs caudodorsally (Fig. 2). The sylvian fissure and limbs delineate the sylvian gyrus's border and its respective sections. The rostral limb delineates the rostral sylvian gyrus, the middle one intersects the middle sylvian gyrus, and the caudal limb marks the border between the middle and the caudal sylvian gyrus (Fig. 2).

### Presylvian sulcus

Our findings show that the presylvian sulcus extends rostroventrally on the lateral hemisphere. This sulcus originates within the frontal lobe and is connected to the sylvian fissure caudally. It is situated dorsolaterally to the rhinal sulcus and delineates the dorsal border of the prorean gyrus as it runs rostrally until it meets the cruciate sulcus (Fig. 2).

### Rhinal sulcus

The rhinal sulcus is the most ventrolateral sulcus in the camel’s cerebral hemispheres. The rhinal sulcus and the olfactory fissure outline the olfactory region of the forebrain. The rhinal sulcus has two segments. The first segment runs as a direct extension of the olfactory fissure, curving slightly dorsally before merging with the presylvian sulcus before encountering the sylvian fissure. The second segment called the caudal rhinal sulcus, begins around the pyriform lobe and extends caudally. This segment was left unmentioned in previous camel brain research, highlighting the novel insights provided by our findings (Fig. 2).

### Ectosylvian sulcus

The ectosylvian sulcus has a deep and curved path within the lateral hemisphere. It is located ventral to the suprasylvian sulcus. It tends to make a caudal and rostral arch around the sylvian fissure (Fig. 2). Our observations revealed that this sulcus has three limbs, each with distinct orientations. The rostral limb begins from the middle ectosylvian sulcus and runs slightly parallel and caudally to the rostral limb of the suprasylvian sulcus. The middle limb is ventrally positioned relative to the suprasylvian sulcus' middle limb. Interestingly, the caudal limb has a longer tract and is unconnected to the middle limb. It runs perpendicularly in a curvy manner ventral to the caudal branch of the suprasylvian sulcus. We observed the sulcus is situated between the caudal sylvian gyrus and the caudal ectosylvian gyrus (Fig. 2).

### Suprasylvian sulcus

It is a large and deep sulcus that roughly separates the cerebral surface into dorsal and lateral sections. It is located dorsal to the ectosylvian and Sylvian sulci. It extends rostrocaudally in an undulating pattern. We observed that the suprasylvian sulcus of the Dromedary camel's brain subdivides into three distinct segments. These have been appropriately named the rostral, middle, and caudal portions, each with distinct characteristics (Fig. 2). The rostral limb of the suprasylvian sulcus begins laterally and runs dorsally until it merges with the middle limb. The latter runs rostrocaudally at the middle of the cerebral hemisphere, and the caudal limb runs until it reaches the occipital lobe. The caudal limb of the suprasylvian sulcus runs parallel to the marginal sulcus (Fig. 2).

### Diagonal sulcus

The diagonal sulcus is situated dorsolaterally to the presylvian sulcus. It begins close to the coronal sulcus and runs caudoventrally toward the sylvian fissure. The diagonal sulcus defines the dorsal border of the diagonal gyrus (Fig. 2).

### Ectomarginal sulcus

In all examined cerebral hemispheres, the ectomarginal sulcus displays a dual-component portion. The caudal part of the sulcus is situated ventrolateral to the marginal sulcus; the other portion is positioned almost dorsolateral to the suprasylvian sulcus. Following a caudal extension, the sulcus is interrupted before resuming a shorter path, aligning almost parallel to the caudal limb of the suprasylvian sulcus (Figs. 1 and 2).

### Marginal sulcus

Our investigation identified the marginal sulcus as the most prominent and distinct sulcus visible on the dorsal surface. It has a considerable depth and runs parallel to the longitudinal fissure. It begins at the middle of the hemisphere and runs further caudally in a wavy fashion until the occipital pole (Figs. 1 and 2).

### Endomarginal sulcus

**Figure 3:**
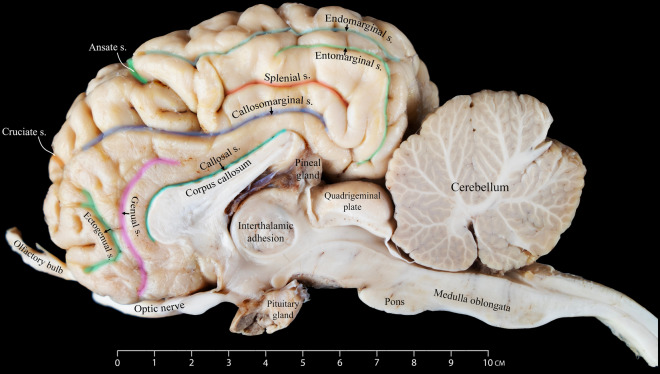
Medial view of the right dromedary cerebral hemisphere, illustrating the intricate arrangement of sulci on the medial surface.

**Figure 4:**
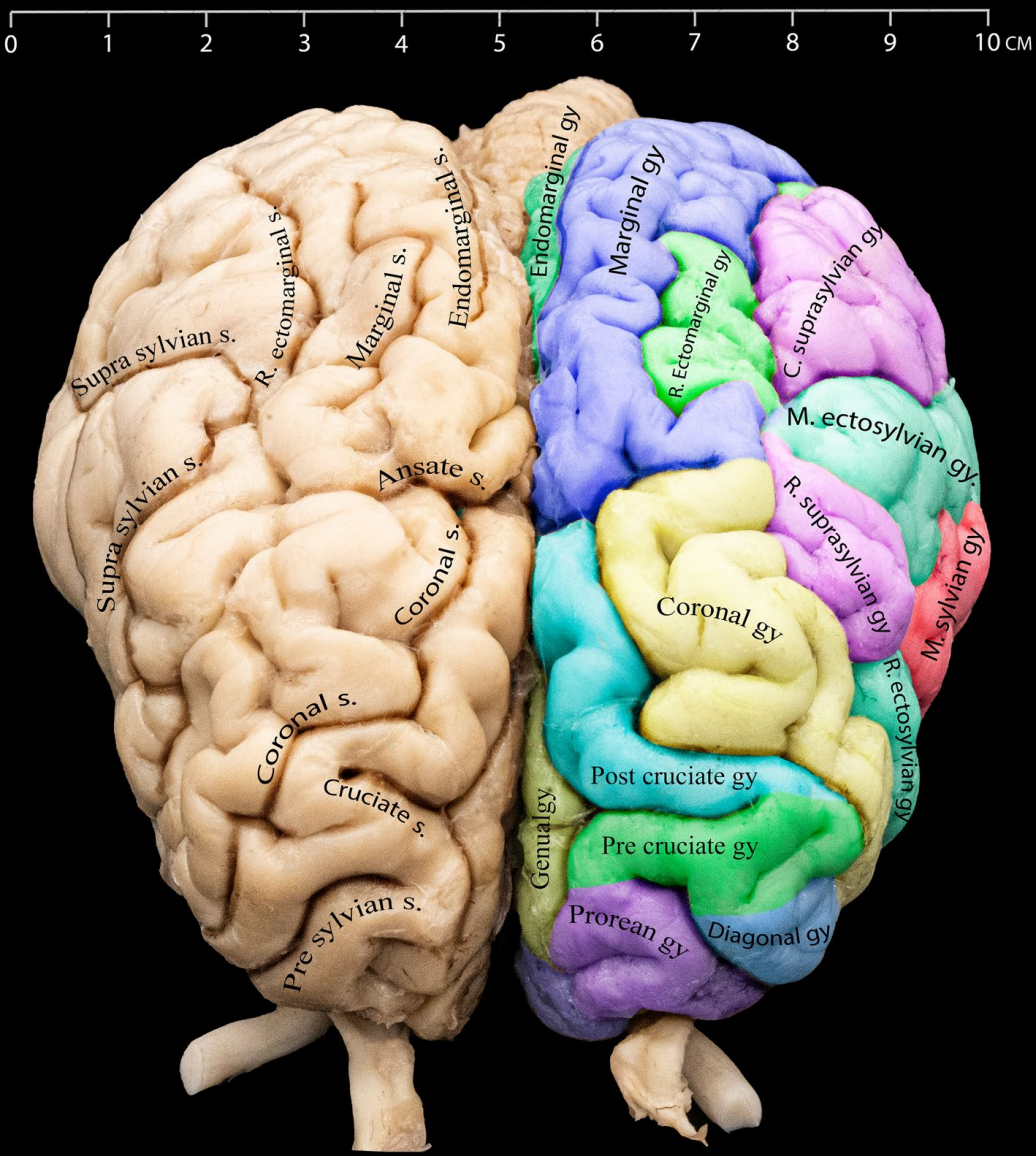
Different gyri and sulci of the cerebral hemispheres as seen from the rostrodorsal view.

Our investigation identified the endomarginal sulcus as a thin sulcus positioned in the hemisphere's most caudodorsal region. It shows a brief stretch parallel to the longitudinal fissure, forming the most medial structure relative to the longitudinal fissure within the cerebrum's caudal region. It is flanked laterally by the caudal portion of the marginal sulcus (Figs. 1, 3, and 4).

### Ansate sulcus

The ansate sulcus is a deep groove with a relatively short span. It is located dorsally, nearly at the hemisphere's midpoint. It extends laterally from the longitudinal fissure. Along its tract, it meets the coronal sulcus rostrally and the ectomarginal sulcus on its lateral end (Figs. 3 and 4).

### Coronal sulcus

The coronal sulcus emerges dorsally, around the frontal lobe. It projects in a rostral and lateral route, thereby defining the lateral boundary of both the post-cruciate and pre-cruciate gyri. In its caudal direction, the coronal sulcus intersects with the ansate sulcus (Figs. 1, 2, and 4).

### Cruciate sulcus

Our investigation revealed that the cruciate sulcus is located dorsally in the most rostral part of the hemisphere. This sulcus, characterized by its relatively short course, extends transversely towards the longitudinal fissure. It emerges rostrally around the midpoint of the coronal sulcus. It is located between the post-cruciate gyrus, which is found caudally to the sulcus, and the pre-cruciate gyrus, positioned rostrally (Figs. 3 and 4).

In the medial view of the dromedary camel cerebrum, we observed numerous sulci displaying diverse features. These sulci vary in characteristics, ranging from delicate and superficial to complex structures with multiple branches. The prominent structure in this view is the corpus callosum, which connects the right and left cerebral hemispheres (Fig. 3). In our study, we meticulously examined and described the various sulci in the medial aspect, as reported below.

### Callosomarginal sulcus

We identified the callosomarginal sulcus as one of the most prominent structures recognizable in the medial view of the dromedary brain. The callosomarginal sulcus is characterized by its considerable length and appearance as a medial extension of the cruciate sulcus, extending caudally with a wavy pattern. The middle segment of this sulcus is situated dorsally and almost parallel to the callosal sulcus. Caudally, the callosomarginal sulcus eventually meets with the entomarginal sulcus (Fig. 3).

### Entomarginal sulcus

The entomarginal sulcus, situated dorsocaudally, and the endomarginal sulcus make up the endomarginal and entomarginal gyri. Initially, this sulcus follows a short and straight route before taking a sharp ninety-degree turn in a ventral direction towards the tectum. The endomarginal sulcus is a shorter structure in a dorsal position relative to the entomarginal sulcus (Fig. 3).

### Callosal sulcus

Our study revealed that this sulcus delineates the corpus callosum, differentiating it from the adjacent cingulate gyrus. The callosal sulcus contributes to forming the deeper portion of the longitudinal fissure (Fig. 3).

### Genual sulcus

As observed in our study, the genual sulcus runs in a rostral direction parallel to the callosal sulcus. It is situated between the genual and the cingulate gyrus. Just before the ascending segment of the genual sulcus, we identified the ectogenual sulcus, which borders the ectogenual gyrus (Fig. 3).

### Cerebral gyri

**Figure 5:**
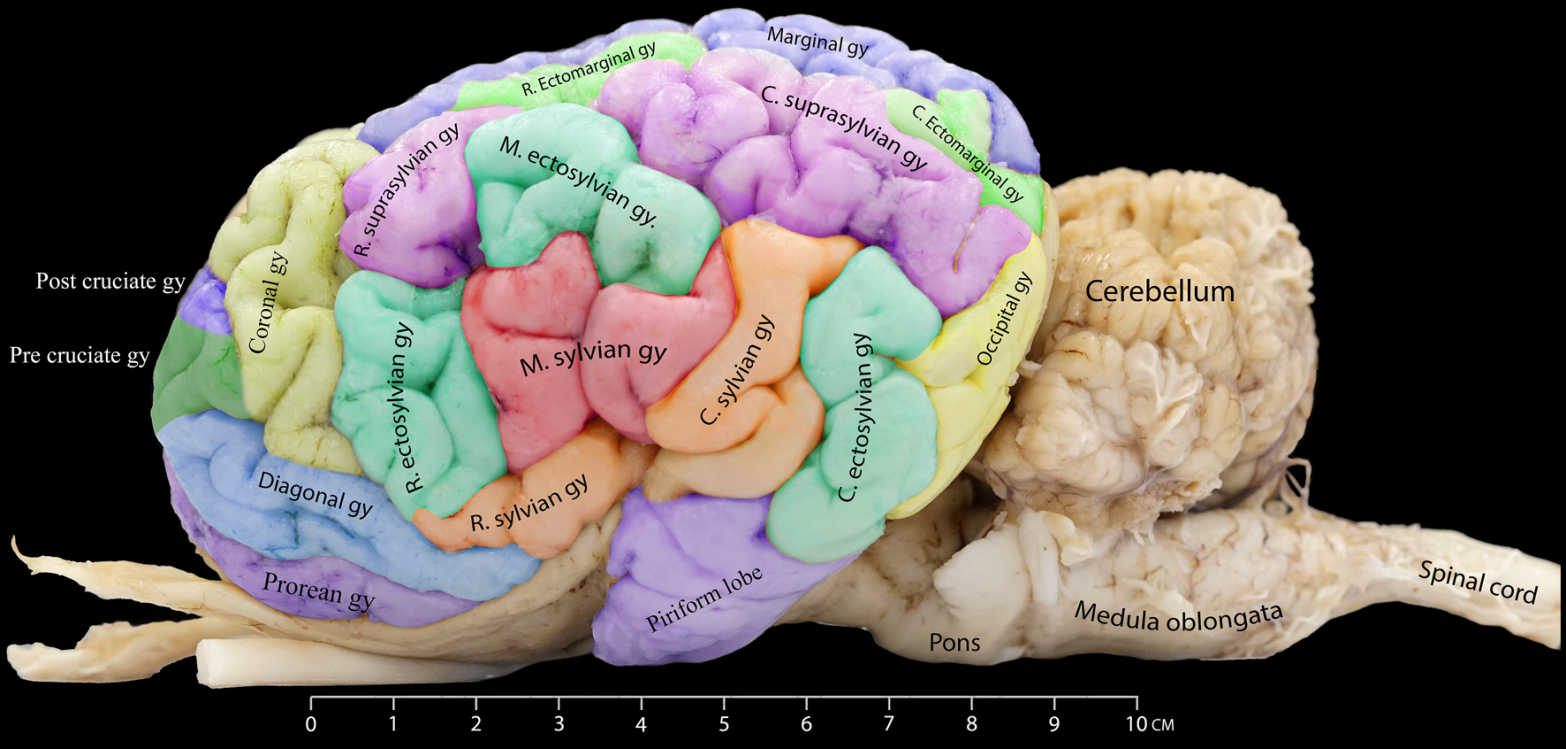
Detailed illustration of the gyri on the lateral surface of the Dromedary camel brain.

**Figure 6:**
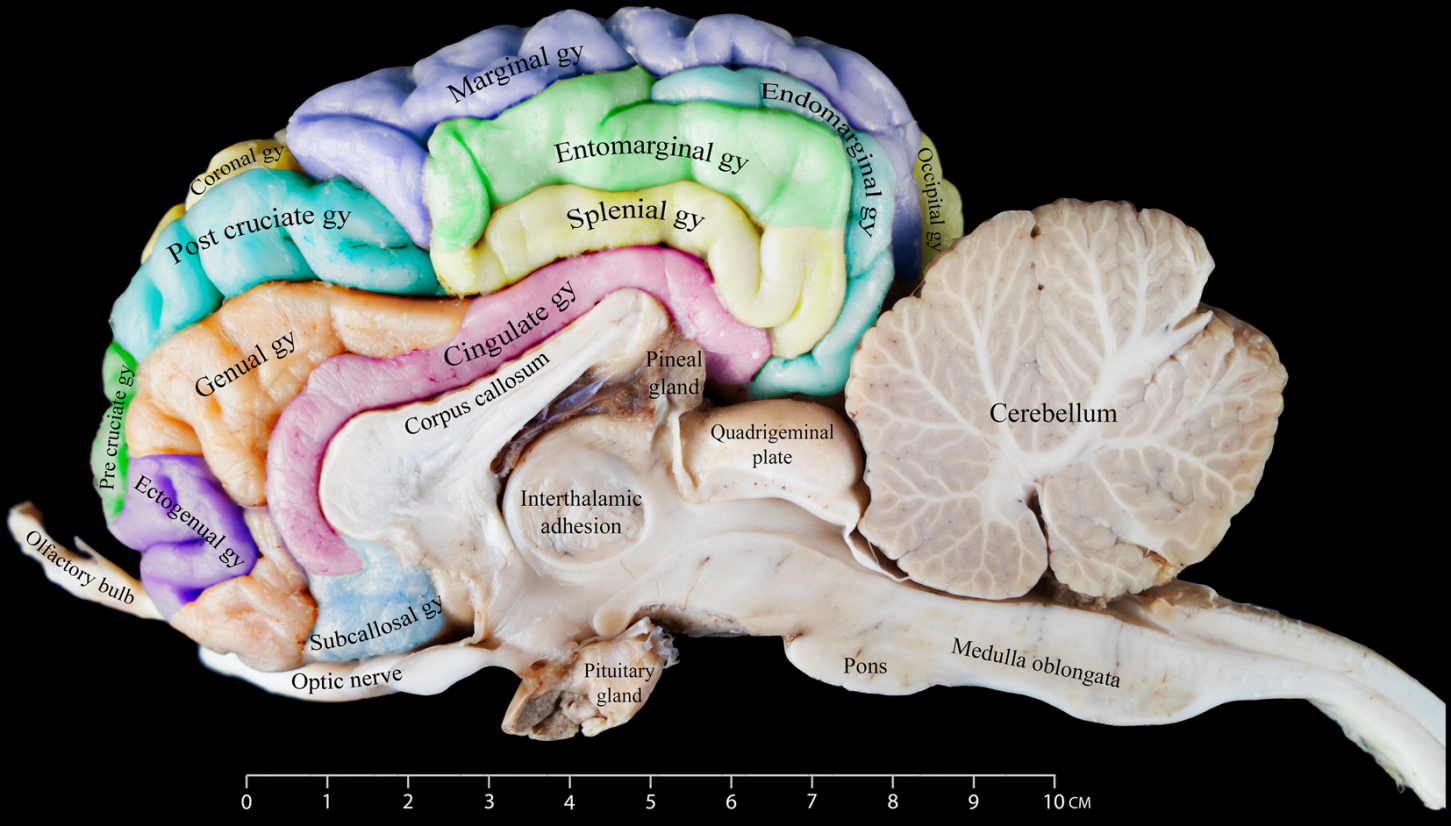
Medial view of the right dromedary cerebral hemisphere, illustrating the intricate arrangement of gyri on the medial surface.

In our study, we precisely identified, described, and characterized the cerebral gyri in the dromedary camel's brain, presenting our findings through detailed figures capturing multiple view aspects, providing a comprehensive visualization of these gyri within the cerebral structure (Figs. 1, 4, 5 and 6).

### Sylvian gyrus

Our study found the sylvian gyrus located dorsal to the olfactory tract and piriform lobe. It has three parts, namely the rostral, middle, and caudal parts, surrounded by the ectosylvian sulcus. The rostral part is separated from the middle part by the rostral ramus of the sylvian fissure. The middle sylvian gyrus is separated from the caudal part by the caudal ramus of the sylvian fissure. The middle ramus of the sylvian fissure is located right above the lateral fissure and divides the middle sylvian gyrus into two parts. The rostral and caudal parts entirely encompass the insular region of the cerebrum (Fig. 5).

### Ectosylvian gyrus

Our findings show that the ectosylvian gyrus is located below the suprasylvian gyrus and has three parts: the rostral, middle, and caudal. The rostral part meets the coronal gyrus from its rostral end. The middle part is surrounded by the rostral suprasylvian gyrus from the rostral margin and the caudal suprasylvian gyrus from its caudal end. The caudal part of the ectosylvian gyrus meets the occipital gyrus and the caudal sylvian gyrus from its caudal and rostral margins, respectively (Fig. 5).

### Suprasylvian gyrus

The suprasylvian gyrus is subdivided into two distinct rostral and caudal sections. These parts are completely seen from the lateral view and separated by the middle part of the ectosylvian gyrus. The rostral part is located caudal to the coronal gyrus, and the caudal part meets the marginal and endomarginal gyri dorsally and the occipital gyrus from its caudal end (Figs. 4 and 5).

### Occipital gyrus

The occipital gyrus, located in the caudal portion of the occipital lobe, is situated beneath and caudal to both the caudal part of the suprasylvian gyrus and ectomarginal gyrus (Figs. 5 and 6).

### Ectomarginal gyrus

Our observations reveal that the ectomarginal gyrus is situated laterally to the marginal gyrus, composed of rostral and caudal parts. These two sections are seen to converge laterally with the caudal portion of the suprasylvian gyrus (Figs. 1 and 5).

### Marginal gyrus

The marginal gyrus is located laterally to the longitudinal fissure of the cerebrum. It extends from the end of the post-cruciate gyrus or the ansate sulcus to the caudal end of the cerebrum (Figs. 1, 4, 5, and 6). Starting from the caudal third of each cerebral hemisphere, a thin endomarginal gyrus (Figs. 1, 4, and 6) intervenes between the marginal gyrus and the longitudinal fissure and extends until the transverse fissure. Both gyri are more prominent in the dorsal view, even though some parts can be observed on the medial aspect of the hemispheres. From its lateral end, the marginal gyrus separately encompasses the rostral and caudal parts of the ectomarginal gyrus (Figs. 1 and 5).

### Post-cruciate gyrus

This gyrus is found caudal to the cruciate sulcus and is separated from the marginal sulcus by the ansate sulcus. It is dorsocaudal to the pre-cruciate gyrus. Unlike the pre-cruciate gyrus, a significant part of this gyrus can be seen on the medial side, above the genual gyrus (Figs. 1, 4, 5, and 6).

### Pre-cruciate gyrus

Our findings show that this gyrus is caudal to the prorean gyrus and rostral to the cruciate sulcus. The rostral view reveals most of this gyrus, with more minor remnants visible in both the medial and lateral views (Figs. 1, 4, 5, and 6).

### Coronal gyrus

In the anterodorsal region of the brain, the coronal gyrus can be found between the longitudinal fissure and the coronal sulcus. It is located lateral to the post-cruciate sulcus, and from a lateral perspective, it extends up to the edge of the diagonal gyrus. From its rostral border, the coronal gyrus meets the post and pre-cruciate gyri. This gyrus is partially seen from the medial view (Figs. 1, 4, 5, and 6).

### Prorean gyrus

It is the most rostral gyrus positioned at the base of the frontal lobe. It is bordered dorsally by the presylvian sulcus. It can be fully seen from the rostral view. A part of the prorean gyrus is located on the lateral side, dorsal to the olfactory nerves (Figs. 4 and 5).

### Diagonal gyrus

This gyrus is dorsal to the prorean gyrus, between the diagonal and presylvian sulci. The diagonal gyrus marks the caudal transition from the olfactory area to the large piriform lobe of the rhinencephalon (Figs. 4 and 5).

### Cingulate gyrus

Positioned within the medial aspect of the dromedary hemisphere, the cingulate gyrus is found immediately surrounding the corpus callosum, the part of the brain where the left and right cerebral hemispheres meet and communicate. The callosal sulcus separates the cingulate gyrus from the corpus callosum. The rostral half of the cingulate gyrus is separated from the genual gyrus by the genual sulcus, while the caudal half is separated from the other gyri found dorsally by the callosomarginal sulcus (Fig. 6).

### Subcallosal gyrus

Our findings revealed that the subcallosal gyrus is located at the rostral extremity of the cingulate gyrus, directly beneath the genu of the corpus callosum. The rostral part of the subcallosal gyrus is defined from the ventral part of the genual gyrus by the genual sulcus (Fig. 6).

### Genual gyrus

The genual gyrus surrounds the cingulate and subcallosal gyri rostrally. It exhibits a wide dorsal part that gradually tapers towards the ventral portion of the gyrus. The ventral part of this gyrus is wider than the middle part. The post-cruciate gyrus can be found here surrounding the genual gyrus dorsally. The most rostral part of the genual gyrus meets the pre-curciate gyrus, which is better seen from the lateral view. The middle-tapered part of the genual gyrus adjoins the ectogenual gyrus (Fig. 6).

### Entomarginal gyrus

The entomarginal gyrus is a compact gyrus found above the splenial gyrus and below the marginal gyrus rostrally and the endomarginal gyrus caudally (Fig. 6). It is a gyrus presented horizontally along the dorsocaudal portion of the medial cerebral hemisphere.

### Splenial gyrus

The splenial gyrus is a medially situated structure located between the entomarginal gyrus dorsally and the caudal portion of the cingulate gyrus ventrally (Fig. 6). It is found around the caudodorsal portion of the medial surface of the brain.

## Discussion

Comprehensive morphological studies of the dromedary brain, particularly its gyri and sulci, are notably lacking compared to the extensive information available for other animal species. In this study, we mapped the sulci and gyri of the camel brain based on previous neuro-anatomical studies^9,10,12,13^, incorporating contemporary anatomical nomenclature^11^. The gyral boundaries were determined by analyzing the arrangement and interrelationships of adjacent sulci. Additionally, we further refined our gyral identifications using cerebral arterial branches^3,14,15^. While a few earlier studies have tentatively explored the external architecture of the camel brain, inconsistencies can be observed between their findings^9,10^. For example, Xie and Wang^10^ identified the orbital gyrus, while Kanan^9^ labeled the same structure as the prorean gyrus. Additionally, Xie and Wang^10^ mentioned the gyrus post-cruciatus, which Kanan^9^ referred to as the posterior sigmoid gyrus. Likewise, the dorsal cingulate gyrus, as described by Xie and Wang^10^, corresponds to the fornicalus gyrus, as labeled by Kanan^9^. Both studies did not comprehensively describe all sulci and gyri of the camel brain; each presented partial accounts of sulci and gyri of the camel brain. Kanan^9^ sometimes employed human terminology, such as naming the sylvian fissure as the central sulcus, a term used in human anatomy.

Consequently, our study provided a comprehensive colored-coded atlas of the brain morphology of the one-humped camel (*Camelus dromedarius*), specifically focusing on describing and identifying its gyri and sulci. Our study established a solid foundation for further research in this area, bridging the gaps left by previous literature.

Our analysis revealed subtle variations in brain anatomy among the samples studied, particularly in the size and folding patterns of gyri and the depth of sulci in individual specimens. These findings align with the observations made by Tecirlioğlu^17^, Thompson^18^, Taner^19^, and Kurt^20^, suggesting that the sulci and gyri on the left and right hemispheres of the same animal species can differ in terms of their shapes and numbers.

The camel brain shares features with other mammals, displaying typical ungulate characteristics, such as the neocortex's intense gyrification^10^. These authors also highlight that while the camel brain's weight is relatively lighter than that of horses and water buffaloes, it is still heavier than that of certain cattle species^10^. The camel brain's exterior structure resembles an equine species more than any other species^9^. However, the camel’s brain has less folding than that of horses, characterized by intense gyrification and cortical thickness^21^.

While the brain of the camel shares several similarities with that of the equines^9^, it also reveals a combination of convolutional patterns from two different suborders of Ungulata, namely, Artiodaclyla and Perissodactyla^9,22^.

Although the camel neopallium was highly heterogeneous in its surface architecture, certain sulci were characteristic in their length, depth, and course, indicating that they were structurally distinct, constant, and almost invariable. These landmarks serve as a reliable guide for topographic orientation. With the help of our thorough study, we could clearly and topographically describe the distinct chain of sulci that runs throughout the whole stretch of the camel neopallium from all perspectives (lateral, medial, and dorsal views).

Our findings indicate that the sylvian fissure of the dromedary brain, commonly referred to as the lateral sulcus^9^, is a prominent groove that defines the boundaries between the temporal, frontal, and parietal lobes. The sylvian fissure displays distinct characteristics as it ascends from the temporal lobe dorsally, including a shorter length, relatively narrow width, and a more linear trajectory. This pattern of the sylvian fissure is also observed in horses^23^. In contrast, the sylvian fissures in ox are considerably deeper and nearly reach the middle of the lateral hemisphere^22^.

Based on our findings, the sylvian fissure gives three limbs: the rostral, middle, and caudal ramus. The middle ramus of the sylvian fissure follows a relatively straight pathway, extending from the sylvian fissure toward the top of the brain. This exhibits a parallel pattern in other ruminants, such as bovines and ovines^22,24^. This sulcus in horses displays a more curved shape resembling the Greek letter "omega" and is particularly deep and prominent^23^. We have observed that the central sulcus described by Kanan^9^ is, in fact, the middle ramus of the sylvian fissure. Hence, the term ‘middle ramus of the sylvian fissure’ better describes the location and pathway of this sulcus, aligning it with the nomenclature used in humans.

Our study revealed that the rhinal sulcus in camels' cerebral hemispheres is positioned as the most ventrolateral sulcus. We found that the rhinal sulcus is divided into two segments. The first segment originates directly from the olfactory fissure and has a slight dorsal curve before merging with the presylvian sulcus and the sylvian fissure. The second segment, the caudal rhinal sulcus, starts at the pyriform lobe and extends caudally (Fig. 2). It is worth noting that previous studies on camel brain anatomy did not extensively explore this particular area, highlighting its overlooked significance.

We observed that the suprasylvian sulcus was distributed extensively over the laterodorsal surface of the cerebral hemisphere. Camels have a notably shallow and simpler suprasylvian sulcus, whereas horses have a deeper suprasylvian sulcus with several branches^21^.

Our investigation found that the dorso-caudal portion of the hemisphere's surface is distinguished by a collection of three sulci, namely the marginal, ectomarginal, and endomarginal sulcus, which are positioned side by side. Among them, the marginal sulcus is positioned centrally, flanked by the ectomarginal sulcus on its lateral side and the endomarginal sulcus on its medial side (Fig. 1). However, in certain specimens, we observed that the caudal segment of the marginal sulcus varied in length and course, making it unpredictable. It either followed a slightly longer lateral course towards the caudal side of the hemisphere, or it occasionally encountered an interruption near its dorsocaudal pole, failing to extend continuously toward the caudomedial surface. This observation contradicts the terminology used by Kanan^9^, where what we now identify as the ectomarginal sulcus was referred to as the parietooccipital fissure. This sulcus should be named "ectomarginal sulcus" due to its location and after comparing it to those of other animal species, providing a more accurate representation of this sulcus in the dromedary brain^11^.

In our study, the detailed names of the gyri were given according to the position and continuity of the sulci that separate them. The sylvian gyri are divided into anterior, dorsal, and posterior parts, similar to the nomenclature used in humans^9^, while we used the terms rostral, middle, and caudal based on the N.A.V^11^ to name the sulci and gyri found on the lateral surface of the dromedary brain.

The ectosylvian and suprasylvian gyrus in horses had more complex structures with more prominent gyri and sulci, and subregions compared to the same region in the camel brain^25,26^. The occipital gyrus in horses is particularly well-developed and has many branches and subregions, while in camels, it appears less complex^21,23^.

According to Kanan^9^, the prorean gyrus is well-marked and is located on the dorsorostral border of the frontal lobe. However, in our research, it was found that it is located lateroventrally above the olfactory nerve.

Our analysis revealed notable discrepancies in the nomenclature used for sulci and gyri observed in the medial view of the camel’s brain as described in the existing literature. Specifically, we found that Kanan^9^ referred to the genual gyrus as the gyrus fornicatus, suggesting that previous studies may have applied human nomenclature when labeling certain sulci and gyri in the camel's cerebral hemisphere^9,10^. The callosomarginal sulcus was identified by Xie and Wang^10^ in their study as being placed dorsal to the actual callosomarginal sulcus, which encircles the cingulate gyrus as observed in our study (Fig. 6). To ensure consistency with the terminology used to name this sulcus in other animals, as reported in other studies, we have revealed that the name splenial sulcus is more precise. According to our findings, a component that Xie and Wang^10^ referred to as the entogenual sulcus is better defined as being a part of the callosomarginal sulcus. We also suggest reclassifying the gyrus that was previously known as the orbital gyrus. We have named it the ectogenual gyrus because of its proximity to the ectogenual sulcus (Fig. 6).

Our study also challenged the terminology used to indicate the dorsal and ventral cingulate gyri as the two separate portions of the cingulate gyrus^10,27^. Based on our observations, we use the term cingulate gyrus to refer to what Xie and Wang^10^ refer to as the ventral cingulate gyrus. We have discovered that the area referred to by Xie and Wang^10^ as the dorsal cingulate gyrus is the entomarginal gyrus (Fig. 6). The latter terms are consistent with the descriptions given by Kanan^9^ and Blanco^27^ in addition to other research on many animal species^22–24,28^, which we believe provides a clearer anatomical representation of these gyri.

In conclusion, our research offers a comprehensive investigation into the gyri and sulci of the dromedary brain, a relatively understudied area in the literature. Our research identified previously overlooked components and enhanced the descriptions of specific structures like the ectomarginal sulcus, splenial sulcus, splenial gyrus, and ectogenual gyrus. These findings demonstrate the importance of further exploration in comparative neuroanatomy, emphasizing the need to develop a comprehensive reference dataset for camel brain anatomy.

## Data Availability

The datasets for this study can be made available by the corresponding author without undue reservation.
